# Coronavirus and Paramyxovirus Shedding by Bats in a Cave and Buildings in Ethiopia

**DOI:** 10.1007/s10393-022-01590-y

**Published:** 2022-06-30

**Authors:** Jennifer K. Lane, Yohannes Negash, Nistara Randhawa, Nigatu Kebede, Heather Wells, Girma Ayalew, Simon J. Anthony, Brett Smith, Tracey Goldstein, Tesfu Kassa, Jonna A. K. Mazet, PREDICT Consortium, Woutrina A. Smith

**Affiliations:** 1 One Health Institute and Karen C. Drayer Wildlife Health Center, School of Veterinary Medicine, University of California, Davis, 1089 Veterinary Medicine Drive, VM3B, Davis, CA 95616 USA; 2 Aklilu Lemma Institute of Pathobiology, Addis Ababa University, P.O. Box 1176, Addis Ababa, Ethiopia; 3 Department of Ecology, Evolution, and Environmental Biology, Columbia University, New York, NY 10027 USA; 4 Ethiopian Wildlife Conservation Authority, Ministry of Environment, Forestry and Climate Change, Addis Ababa, Ethiopia; 5 School of Veterinary Medicine, University of California, Davis, Davis, CA 95616 USA; 6 Genome Center & Biomedical Engineering, School of Medicine, University of California, Davis, Davis, CA 95616 USA; 7 Zoological Pathology Program, University of Illinois at Urbana-Champaign, Brookfield, IL 60513 USA

**Keywords:** bat, coronavirus, paramyxovirus, Ethiopia, cave, viral shedding

## Abstract

Bats are important hosts of zoonotic viruses with pandemic potential, including filoviruses, MERS-Coronavirus (CoV), SARS-CoV -1, and likely SARS-CoV-2. Viral infection and transmission among wildlife are dependent on a combination of factors that include host ecology and immunology, life history traits, roosting habitats, biogeography, and external stressors. Between 2016 and 2018, four species of insectivorous bats from a readily accessed roadside cave and buildings in Ethiopia were sampled and tested for viruses using consensus PCR assays for five viral families/genera. Previously identified and novel coronaviruses and paramyxoviruses were identified in 99 of the 589 sampled bats. Bats sampled from the cave site were more likely to test positive for a CoV than bats sampled from buildings; viral shedding was more common in the wet season; and rectal swabs were the most common sample type to test positive. A previously undescribed alphacoronavirus was detected in two bat species from different taxonomic families, sampling interfaces, geographic locations, and years. These findings expand knowledge of the range and diversity of coronaviruses and paramyxoviruses in insectivorous bats in Ethiopia and reinforce that an improved understanding of viral diversity and species-specific shedding dynamics is important for designing informed zoonotic disease surveillance and spillover risk reduction efforts.

**Supplementary Information:**

The online version contains supplementary material available at 10.1007/s10393-022-01590-y.

## Introduction

Bats are frequently identified as hosts of zoonotic viruses worldwide, which has implications for the role of bats as evolutionary hosts of viruses with pandemic potential (Li et al. 2005; Zhou et al. 2020). The emergence of severe acute respiratory syndrome coronavirus (SARS-CoV-1), Middle East respiratory syndrome coronavirus (MERS-CoV), Nipah virus, and most recently, SARS-CoV-2 as major human pathogens likely spilling over from bats highlights the need for surveillance of viruses in wildlife at key interfaces where people could become infected (Yob et al. 2001; Lau et al. 2005; Li et al. 2005; Woo et al. 2006; Vijaykrishna et al. 2007; Anthony et al., 2012a, 2017a, b; Corman et al., 2014a, 2014b, 2020; Kreuder Johnson et al. 2015; Wacharapluesadee et al. 2015, 2021; Letko et al. 2020; Zhou et al. 2020).

Over the last decade, studies across Africa have confirmed that insectivorous and frugivorous bats serve as hosts for a wide variety of viruses from a range of viral families with known and potential impacts on animal and human health (Pourrut et al. 2009; Tong et al. 2009; Markotter et al. 2019; Nziza et al. 2020). Furthermore, other viruses of evolutionary importance and possible concern also continue to be identified, highlighting the potential for hotspots of viral diversity and emerging infectious disease spillover (Han et al. 2016; Allen et al. 2017; Plowright et al. 2017; Kumakamba et al. 2021; Wells et al. 2021). For example, Egyptian fruit bats (*Rousettus aegyptiacus*) are known reservoirs of the Marburg virus (Family *Filoviridae;* Genus *Marburgvirus*) and are found in sites ranging across the African continent, including Sierra Leone, Uganda, DRC, Kenya, South Africa, Gabon, and Zambia (Towner et al. 2007, 2009; Pourrut et al. 2009; Kuzmin et al. 2010; Amman et al. 2012, 2014, 2020; Changula et al. 2018; Paweska et al. 2018; Kajihara et al. 2019). In Sierra Leone and Kenya, different teams of researchers identified Bombali virus (Family *Filoviridae;* Genus *Ebolavirus*) in two species of insectivorous bats, *Chaerephon pumilus* and *Mops condylurus* (Goldstein et al. 2018; Forbes et al. 2019). Other research teams have reported SARS- and MERS-related coronaviruses (CoVs) in Kenyan and Ugandan bat species (*Chaerephon sp., Pipistrellus cf. hesperidus, Hipposideros caffer*) also found in Ethiopia (Tong et al. 2009; Anthony et al. 2017a; Waruhiu et al. 2017; Wells et al. 2021). In Rwanda, a new betacoronaviru*s* and several different paramyxoviruses (PMV) were reported in the insectivorous bat species *Hipposideros ruber, Rhinolophus clivosus*, and *Otomops martiensseni* (Markotter et al. 2019). Another Rwandan study identified two novel SARS-CoV-related coronaviruses (SARSr-CoV) from *Hipposideros ruber* and *Rhinolophus clivosus* co-roosting in caves visited by tourists, as well as *Hipposideros caffer bats* sampled in a national park, in addition to multiple other known and new CoVs (Nziza et al. 2020; Wells et al. 2021). Seasonality of viral shedding in bats has not been extensively studied, but CoV infections with seasonal peaks have been documented in *Hipposideros gigas* and *Hipposideros cf. ruber* bats sampled from caves in Gabon, in multiple species of frugivorous and insectivorous bats at sites across Uganda, Rwanda, Tanzania, and the Congo Basin, and in *Mormopterus francoismoutoui* on Reunion Island in the Indian Ocean (Maganga et al. 2020; Montecino-Latorre et al. 2020; Kumakamba et al. 2021; Joffrin et al. 2022).

Bats are some of the most geographically distributed mammals in the world, and they provide essential ecosystem services for natural and agricultural systems including seed dispersal, pollination, fertilizer production through guano, and insect control (Kunz et al. 2011; Voigt and Kingston 2016). Capable of long-distance flight, different patterns of migration, and species co-habitation, the life history and reproductive patterns of various bat species may also facilitate the exchange of viruses among bat species, as well as with other wildlife species (Calisher et al. 2006). Increasingly around the world, anthropogenic activities involving land-use change, urbanization, mining, and deforestation have led to more contact between humans and wildlife, including bats, leading to increased opportunities and risk for potential viral spillover (Kunz et al. 2011; Johnson et al. 2020).

Ethiopia is home to at least 70 species of bats, the majority of which are insectivorous, including eight species within the Rhinolophidae family (Horseshoe bats) (NABU 2017). Varied habitat availability exists due to Ethiopia’s vast diversity of ecological niches and geographical location between the Arabian Peninsula, sub-Saharan region, and East Africa. While home to a diverse range of bat species, the scientific literature contains very little information on bat ecology and viral diversity in Ethiopia. This study reports on viral shedding risk factors and co-infection in four species of insectivorous bats sampled from buildings and a highly accessible roadside cave in Ethiopia from 2016 to 2018.

## Methods

### Study Area

**Figure 1:**
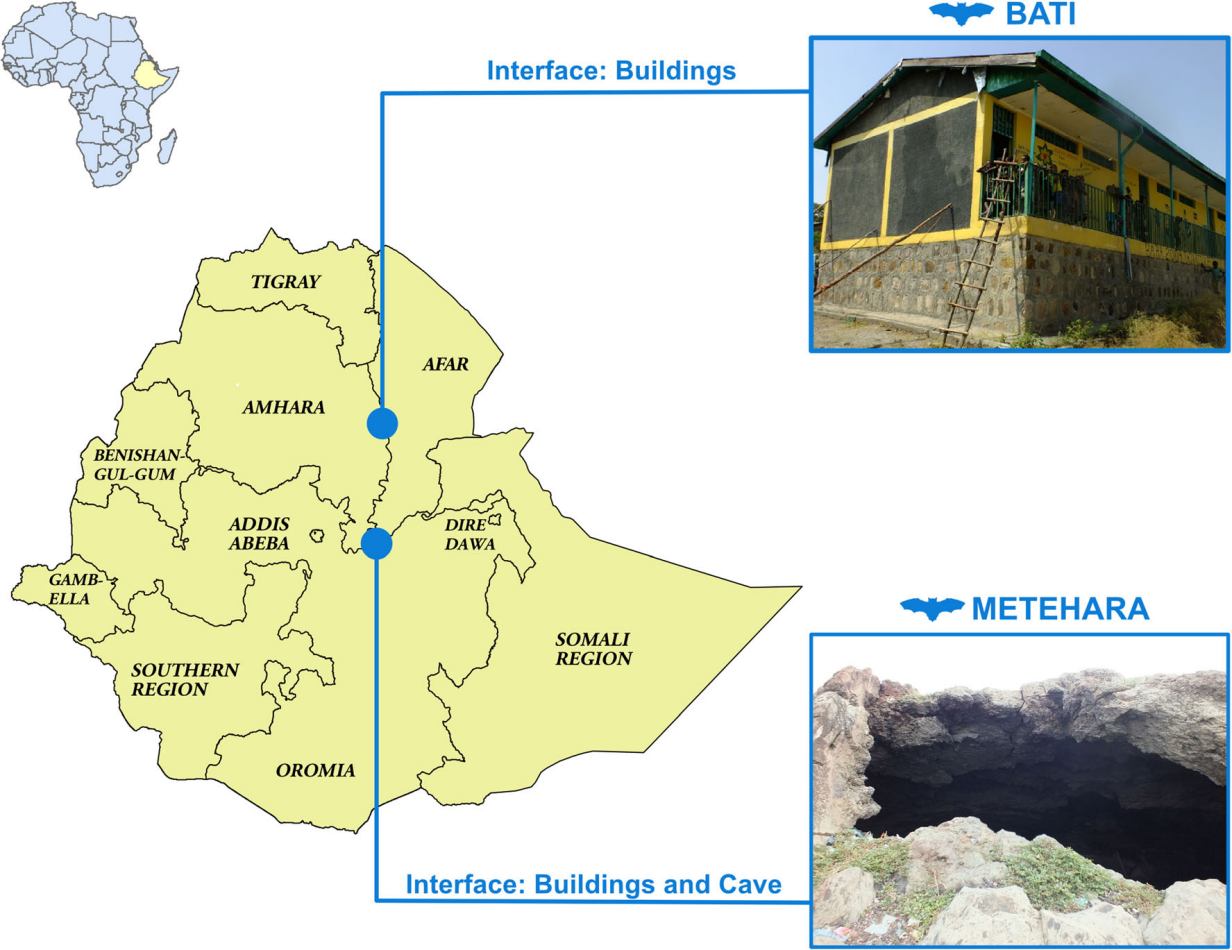
Geographical location of the sampling sites at Bati and Metehara, Ethiopia. Map generated using ArcGIS 10.1 software.

Bat sampling sites were selected in collaboration with the Ethiopian Wildlife Conservation Authority, by identifying high-risk interface study sites in two regions. A high-risk interface was defined as a location where people had high potential to come into contact with bats as well as their excrement and nearby livestock markets and/or wildlife reserves. Study locations included urban and peri-urban dwellings in Metehara town in eastern Ethiopia, a blister cave adjacent to a major highway in Metehara, and in Bati town in north-central Ethiopia. (Fig. 1).

Metehara (8.8995 N, 39.9169 E) is west of Awash National Park and the border of the Afar Regional State on the Addis Ababa-Dire Dawa-Djibouti highway, which is the major transit corridor connecting Ethiopia with the seaport in Djibouti. Volcanic blister caves along the roadside in Metehara are primary habitats for insectivorous bats and are also used as shelter by transient laborers (Personal Communication, PREDICT-Ethiopia team). Water that pools at the bottom of these caves is used as a water source by a local car wash to wash large lorries overnighting along the highway. In Metehara, bats were sampled from the study cave, as well as from roofs of buildings including residences and businesses. Bati town (11.193 N, 40.015 E) is located at an elevation of 1502 m, 417 km northeast of the capital city Addis Ababa in the Oromia zone of the Amhara Region, close to the border with the Afar region, and west of the Mile Serdo Wildlife Reserve. Bati is home to Ethiopia’s largest cattle and camel market and is a major thoroughfare for livestock moving throughout Ethiopia and the greater region. Insectivorous bats roosting in buildings are very common in the area. Bats were sampled from roosts located in the roofs of small businesses, the local primary school, and the local health clinic.

### Bat Capture and Sampling

Between July 2016 and October 2018, 589 bats were captured and released using mist net techniques during wet and dry seasons by trained field personnel (FAO 2011; PREDICT 2016). Season was determined using the georeferenced TerraClimate global climate and precipitation dataset using site location, month sampled, and median precipitation values (Abatzoglou et al. 2018). Sampling events were classified as “wet” season events when precipitation values were greater than or equal to the median of all TerraClimate data for that location from 1998 to 2018; otherwise events were classified as “dry” season. Using this classification system, wet season sampling events occurred in March, June, July, August, and September; dry season sampling events occurred in May and October. This aligned with historical rain fall patterns in parts of Ethiopia, with two distinct wet seasons (March–mid May, end June–September) and a long dry season between October and February (Cheung et al. 2008). Once captured, bats were manually removed from nets, and under gentle manual restraint, species were identified in the field; morphometric evaluation was conducted including age class, sex, body weight, reproductive status; and biometric measurements (e.g., length of forearm, head, and body) were recorded (Menzel et al. 2002). Bat age class (adult, subadult, or juvenile) was determined based on size, morphology, and presence/absence of secondary sexual characteristics (PREDICT 2016). Using sterile polyester-tipped swabs, oral and rectal swabs were obtained from all animals (unless size precluded swabbing). When possible, 2 × 500 μL whole blood samples were also obtained by venipuncture using a non-heparinized syringe or heparinized glass hematocrit tube (PREDICT 2016). All sample types (swabs and blood) were then transferred into two specimen tubes containing 500 μL viral transport medium or 500 μL TRIzol reagent. The samples were immediately transferred into liquid nitrogen in the field for transportation and stored until testing in – 80°C freezers in the laboratory. A veterinarian was present for all sampling events, and all animals were safely released within three hours at the site of capture. During all animal sampling events, minimum required personal protective equipment for personnel included eye protection, N-95 respirators, nitrile gloves, Tyvek suits, and shoe covers.

### Biological Sample Analysis

Total RNA was extracted using the Direct-Zol Kit (Zymo Research Corp), and cDNA synthesis was performed using Superscript III (Invitrogen). Samples were screened for five viral families including corona-, filo-, paramyxo-, flavi-, and influenza viruses using broadly reactive consensus conventional polymerase chain reaction (PCR) assays with positive and negative controls (Moureau et al. 2007; Zhai et al. 2007; Tong et al. 2008; Quan et al. 2010; Watanabe et al. 2010; Anthony et al. 2012b, 2015). Briefly, the assays targeted: the exonuclease (nsp14) of the orf1ab gene (Quan et al. 2010) and the RNA-dependent RNA polymerase (RdRp) of the orf1ab gene (Watanabe et al. 2010) for coronaviruses; the polymerase (L) gene for paramyxoviruses (Tong et al. 2008); the L gene for filoviruses (Zhai et al. 2007); the NS5 gene for flaviviruses (Moureau et al. 2007); and the PB1 gene and M gene for influenza viruses (Anthony et al. 2012b, 2015); all primers used are described in the above cited references. A synthetic plasmid constructed with the binding sites for the specific assays, but otherwise lacking viral sequence was used for the positive control. PCR products of the expected size were cloned (pCR4-TOPO vector; Invitrogen Corp.) and Sanger sequenced (ABI3730 Capillary Electrophoresis Genetic Analyzer; Applied Biosystems, Inc., Foster City, CA). Sequences were edited in Geneious Prime (Version 2019.1.3), uploaded to the NCBI GenBank database (Accession numbers available in Supplemental Table), and compared with known sequences in the GenBank database (Kearse et al. 2012). Sequences were classified into viral taxa by aligning sequences with known reference sequences collected from GenBank followed by manual alignment.

### Bat Species Identification

Bat species field identification was confirmed by host species DNA barcoding using PCR assays targeting fragments of the cytochrome B gene (cytB) and cytochrome oxidase subunit 1 (CO1) mitochondrial genes (Townzen et al. 2008). A threshold of 97% sequence identity was used to confirm the bat species, while sequences with < 95% sequence identity were classified to the genus level only.

### Data Analysis

The data were entered into an encrypted cloud database and extracted, cleaned, summarized, and visualized using Microsoft Excel and R v3.6.0 (Chang 2014; Wickham 2016, 2019a, b; R Core Team 2019; Rudis 2019; Wilke 2019; Firke 2020; Harrison et al. 2020; Lüdecke et al. 2020; Wickham et al. 2020; Wickham and Henry 2020). Fisher’s exact test was used to compare the proportion of samples positive for viral detection by species, sex, age, season, sampling interface, and specimen type. Multivariable logistic regression analyses were conducted to ascertain the association between bats testing positive and their species, age, sex, and the season during which they were sampled. Code for the paper, including figure development is available at https://doi.org/10.5281/zenodo.6032454. Phylogenetic trees including our viral findings were created by aligning sequences using the Geneious alignment algorithm. Best-fit evolutionary models were determined and maximum likelihood statistical support for phylogenetic trees was generated using IQTREE (v.1.6.12) with 100 bootstraps (Minh et al. 2020). All three best-fit evolutionary models were found to be GTR + I + G.

## Results

**Figure 2:**
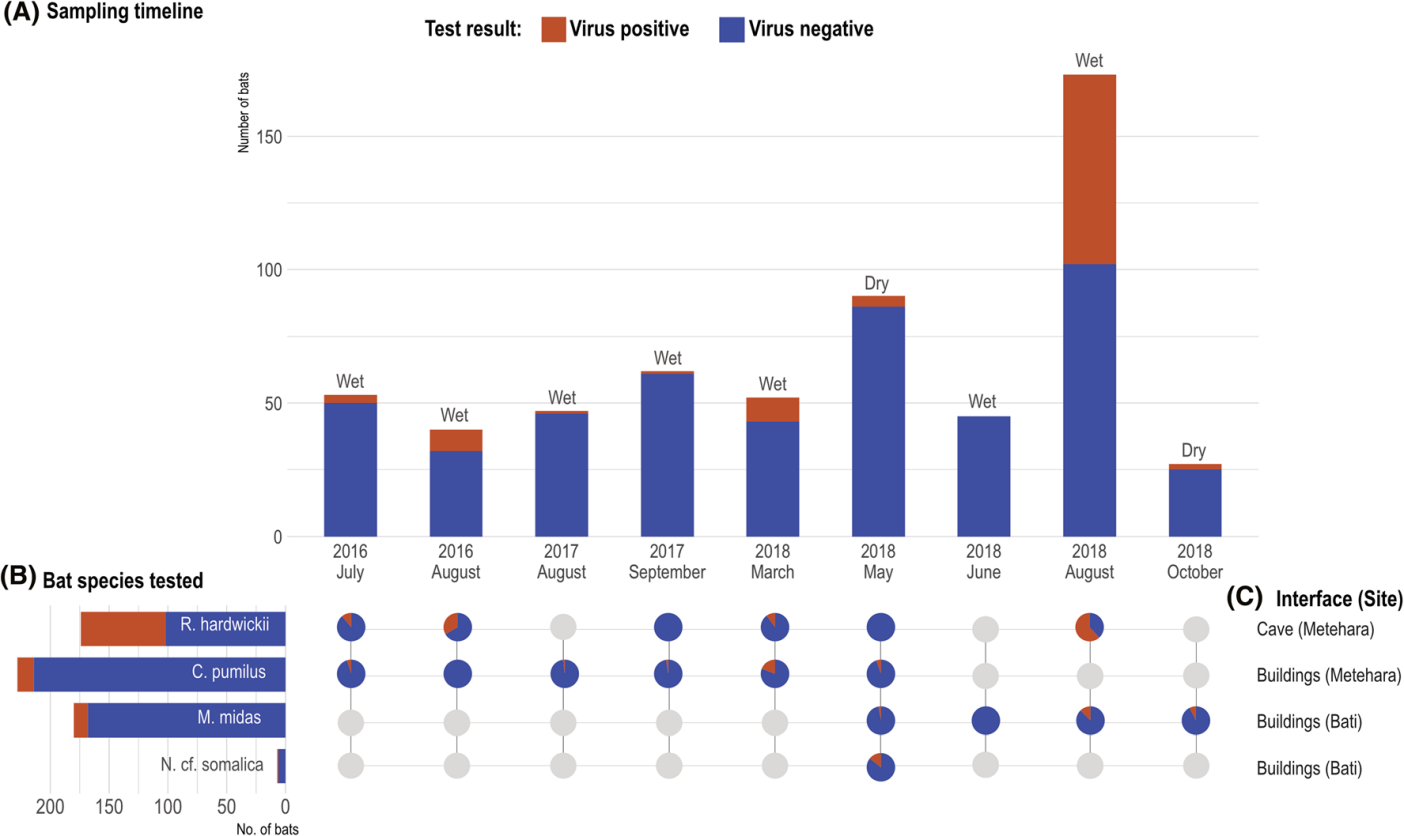
**A** Summary of bat species sampled by sampling event month and season; **B** Species of bats tested and proportion of positive bats at each sampling event; **C** The sampling interface and location. The gray circles depict sampling months and locations when no bats were sampled.

A total of 589 insectivorous bats representing three families and four species of bats were captured and sampled during 12 surveillance events. Eight sampling events occurred at the Metehara site between July 2016 and August 2018; four events occurred at the Bati site between May and October 2018 (Fig. 2). Overall, 68.3% of bats were sampled at the Metehara site and 31.7% of bats were sampled at the Bati site. Bat species sampled at the Metehara site included *Chaerephon pumilus* (Little free-tailed bats, Family: Molossidae; *n* = 228) and *Rhinopoma hardwickii* (Lesser mouse-tailed bats, Family: Rhinopomatidae; *n* = 174). Bat species sampled at the Bati site included *Mops midas* (Midas free-tailed bats, Family: Molossidae; *n* = 180) and *Neoromicia cf. somalica* (Somali serotine bats, Family: Vespertilionidae; *n* = 7) (Table 1).

**Table 1:** Summary of all bats sampled and viral testing results in Metehara and Bati, Ethiopia from 2016 to 2018.

Sampling location	Sampling interface	Taxonomic family	Species name	Total number of bats sampled	Prevalence virus-positive bats % (*n*)	Oral swab % positive (*n*)	Rectal swab % positive (*n*)	Viruses detected (*n*)
Metehara	Building	Molossidae	*Chaerephon pumilus*	228	6% (14)	2% (4)	6% (13)	*Chaerephon* bat coronavirus/Kenya/KY22/2006 (11)
								*Chaerephon* bat coronavirus/Kenya/KY41/2006 (2)
								*Eidolon* bat coronavirus (1)
								PREDICT_PMV-24 (2)
	Cave	Rhinopomatidae	*Rhinopoma hardwickii*	174	41% (72)	17% (29)	39% (67)	*Eidolon* bat coronavirus (1)
								PREDICT_CoV-114 (72)
Bati	Building	Molossidae	*Mops midas*	180	7% (12)	0% (0)	7% (12)	PREDICT_CoV-114 (5)
								PREDICT_PMV-175 (7)
	Building	Vespertilionidae	*Neoromicia cf. somalica*	7	14% (1)	14% (1)	0% (0)	*Eidolon* bat coronavirus (1)
Total				589	17% (99)	6% (34)	16% (92)	

Samples were screened for five viral families including corona-, filo-, paramyxo, flavi-, and influenza viruses using broadly reactive consensus conventional polymerase chain reaction (PCR) assays.

Of the 589 bats tested, 16.8% of bats were positive for one or more virus, with a ‘virus’ loosely defined as a discrete cluster of phylogenetically related sequences. Overall, six different viruses were identified in 99 bats, including four coronaviruses and two paramyxoviruses (Table 1). The previously undescribed viruses detected included a paramyxovirus, referred to as PREDICT_PMV-175, *n* = 7, and an alphacoronavirus, referred to as PREDICT_CoV-114, *n* = 77. Sequences detected that were consistent with previously published sequences in GenBank included: Chaerephon bat coronavirus/Kenya/KY22/2006 (GenBank accession number HQ728486), *n* = 11; Chaerephon bat coronavirus/Kenya/KY41/2006 (GenBank accession number HQ728481), *n* = 2; Eidolon bat coronavirus (GenBank accession number HQ728482), *n* = 3; and PREDICT_PMV-24 (GenBank accession number KP963829), *n* = 2. Whole genome sequencing to confirm these isolates were exact matches to those previously identified was not performed. Three bats had co-infections: one adult female *Rhinopoma hardwickii* tested positive for alphacoronavirus PREDICT_CoV-114 and betacoronavirus *Eidolon* bat coronavirus, while two adult *Chaerephon pumilus* (one male, one female) tested positive for Chaerephon bat coronavirus/Kenya/KY22/2006 and PREDICT_PMV-24. The presence of the same virus in different insectivorous bat families was detected for two different CoVs (PREDICT_CoV-114 and Eidolon bat coronavirus, Table 1). The alphacoronavirus PREDICT_CoV-114 was detected at both interface sites in two bat species from different taxonomic families, all of which were detected in the wet season, with the majority (97.4%) of positives detected in August of 2016 and 2018 (Fig. 2). In Metehara, this virus was detected in 72 (41.4%) *Rhinopoma hardwickii* bats sampled in a blister cave in 2016 and 2018. In Bati, PREDICT_CoV-114 was found in five *Mops midas* bats sampled from buildings in August 2018. No bat samples tested positive for influenza, flavi-, or filoviruses.

**Figure 3:**
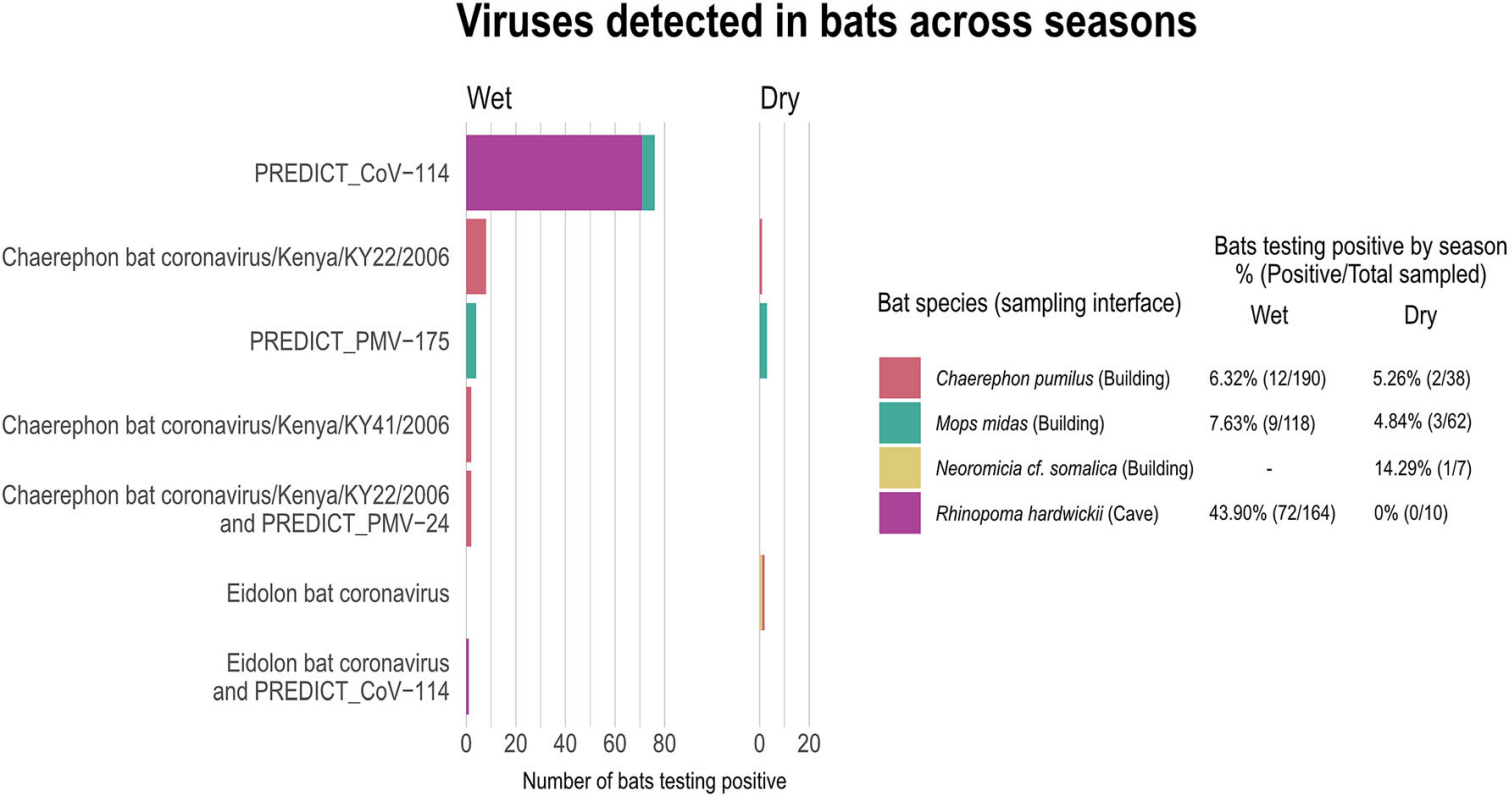
Viral findings by bat species and sampling interface.

Wet season samples represented 80% of the bats sampled, with 472 bats sampled during the wet season; 117 bats were sampled during the dry season (Figs. 2 and 3). More virus-positive bats were detected during the wet season than the dry season (19.7% vs 5.1%; *p* < *0.001)* (Table 2). Bats sampled at the Metehara cave interface were more likely to test positive than bats sampled from building interface sites (41.4%; *n* = 72/174 vs 6.5%; *n* = 27/388);* p* < 0.001). Rectal swabs were the most common specimen type testing positive (15.6%; n = 92/589) as opposed to oral swabs (5.8%, *n* = 34/589) (Table 2). Twenty-seven bats tested positive via both rectal and oral swabs with seven bats testing positive on oral swabs only.

**Table 2:** Viral prevalence in bats by sex, age class, season, species, and specimen type.

		% (Positive/Total)	*P*
Sex	Female Male	16.1% (66/409) 18.3% (33/180)	0.550
Age class	Subadult	30.4% (17/56) 15.4% (82/533)	0.008
	Adult		
Season	Wet	19.7% (93/472) 5.1% (6/117)	< 0.001
	Dry		
Species	*Chaerephon pumilus*	6.1% (14/228)	< 0.001
	*Mops midas*	6.7% (12/180)	
	*Neoromicia cf. somalica*	14.3% (1/7)	
	*Rhinopoma hardwickii*	41.4% (72/174)	
Interface*	Cave	41.4% (72/174)	< 0.001
	Building	6.5% (27/415)	
Specimen type**	Rectal swab	15.6% (92/589)	< 0.001
	Oral swab	5.8% (34/589)	

^*^Cave interface represented only *Rhinopoma hardwickii* bats, while building interfaces included the other three bat species; ** Analysis for specimen type conducted at specimen level; all other analyses conducted at individual animal level.

Multivariable logistic regression analysis was performed for positive viral test results from *Rhinopoma hardwickii, Chaerephon pumilus,* and *Mops midas; Neoromicia cf. somalica* bats were excluded from this analysis due to low sample size. A higher proportion (30.4%; *n* = 17/56) of subadult bats tested positive compared to adults (15.4%; *n* = 82/533); however, this difference was not statistically significant in the multivariable logistic regression analysis (Table 3); all *Mops midas* bats sampled were adults. The odds of a *Rhinopoma hardwickii* bat testing positive were approximately 10 times higher than that for a *Chaerephon pumilus* bat (*p* < 0.001; Table 3). Similarly, bats sampled during wet season had higher odds of testing positive than those sampled during dry seasons (*p* = 0.05).

**Table 3:** Association between bats testing positive for coronaviruses or paramyxoviruses and their species, sex, age, and season in which they were sampled.

	Odds ratio	95% CI	*p*
*Sex*			
Male (v. Female)	1.35	(0.80, 2.28)	0.259
*Age*			
Subadult (v. Adult)	1.26	(0.62, 2.50)	0.522
*Season*			
Wet (v. Dry)	2.67	(1.09, 8.06)	0.050
*Species*			
*Mops midas* (v. *Chaerephon pumilus*)	1.35	(0.59, 3.05)	0.475
*Rhinopoma hardwickii* (v. *Chaerephon pumilus*)	10.39	(5.69, 20.23)	< 0.001

Odds ratio and 95% confidence interval (95% CI) for each predictor in the multivariable logistic regression analysis are presented. *Neoromicia cf. somalica* bats were excluded from this analysis due to low sample size.

**Figure 4:**
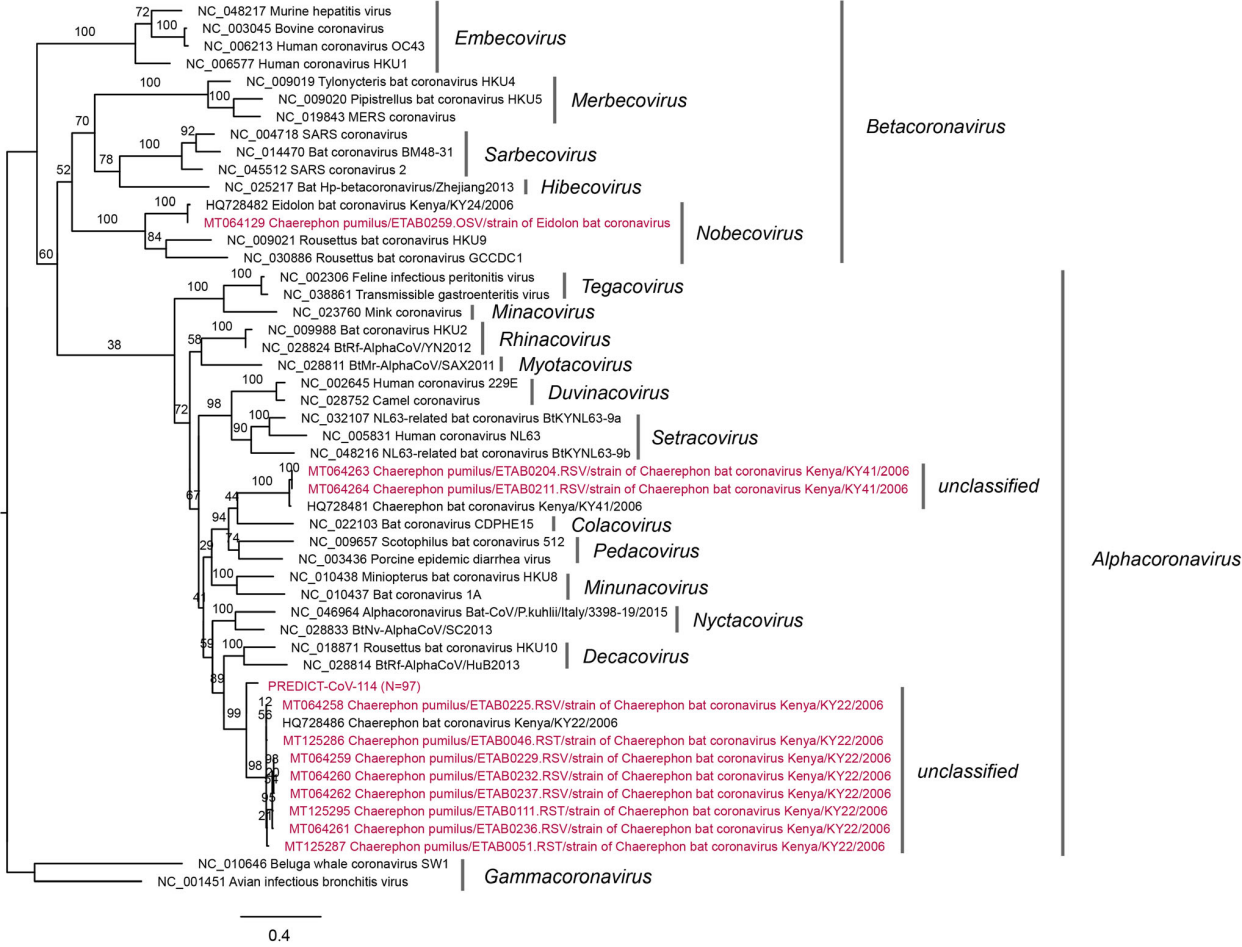
Coronavirus phylogenetic tree constructed based on the exonuclease (nsp14) of the orf1ab gene (Quan et al. 2010). A best-fit evolutionary model was determined, and maximum likelihood statistical support for the phylogenetic tree was generated using IQTREE (v.1.6.12) with 100 bootstraps and a GTR + I + G model. The genus *Betacoronavirus* appears non-monophyletic in this tree; however, bootstrap support for this topology is low, likely because of the short fragment size of the Quan PCR product. Established coronavirus subgenera are annotated immediately to the right of the phylogeny and established genera are annotated at the rightmost side of the figure.

**Figure 5:**
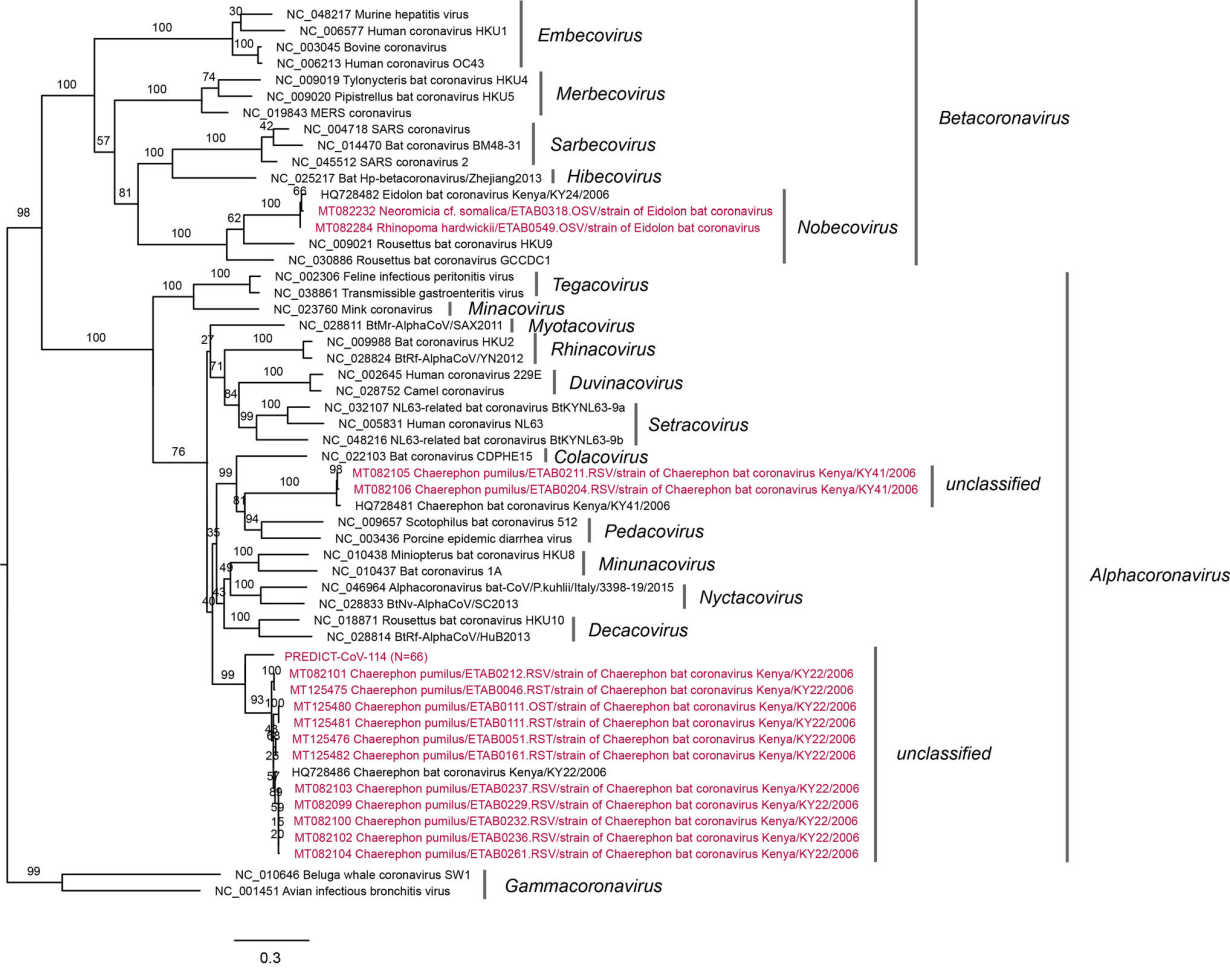
Coronavirus phylogenetic tree constructed based on RNA-dependent RNA polymerase (RdRp) of the orf1ab gene (Watanabe et al. 2010). A best-fit evolutionary model was determined and maximum likelihood statistical support for the phylogenetic tree was generated using IQTREE (v.1.6.12) with 100 bootstraps and a GTR + I + G model. Established coronavirus subgenera are annotated immediately to the right of the phylogeny and established genera are annotated at the rightmost side of the figure.

**Figure 6:**
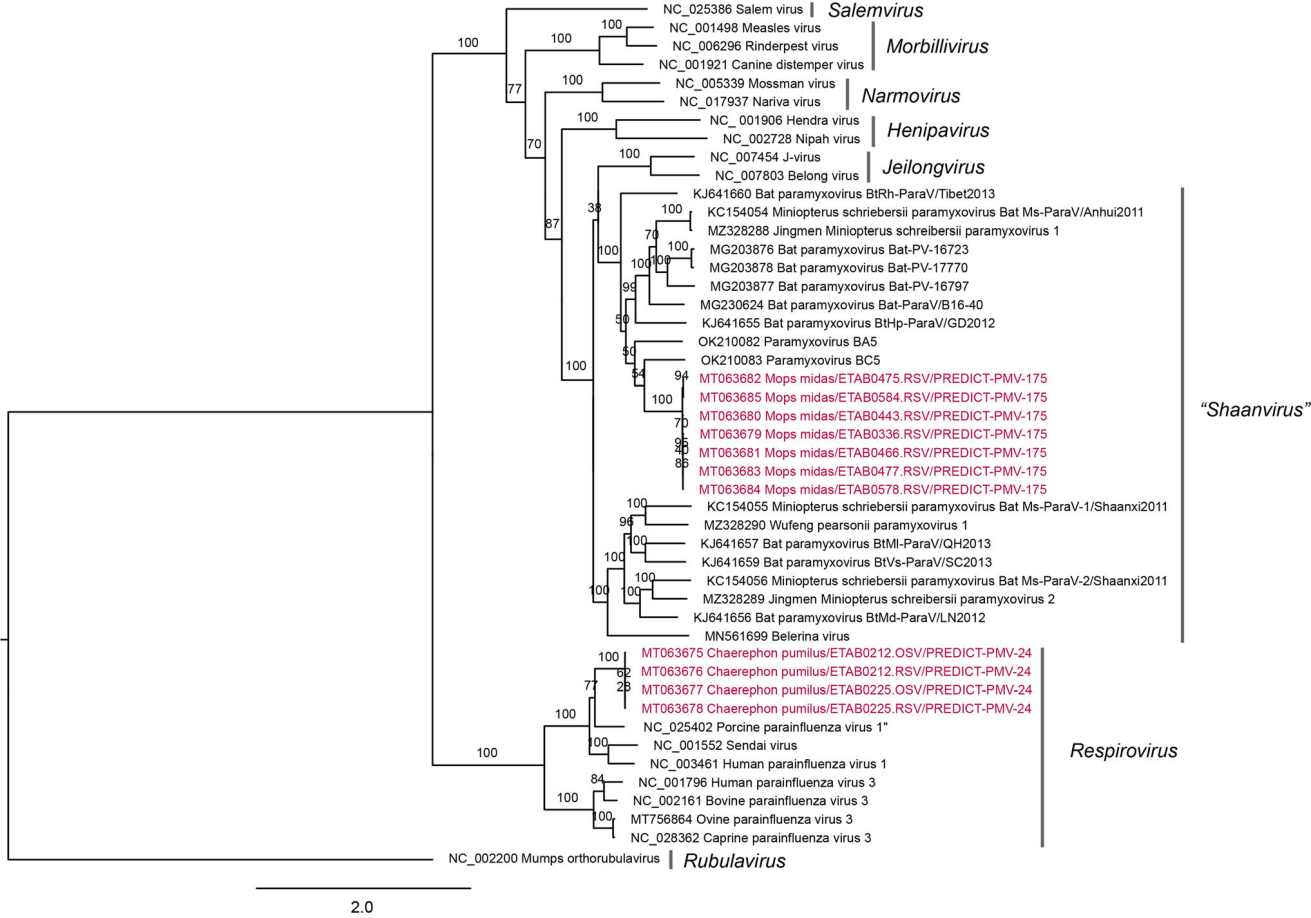
Paramyxovirus phylogenetic tree constructed based on non-overlapping fragments of the polymerase (L) gene (Tong et al. 2008). A best-fit evolutionary model was determined, and maximum likelihood statistical support for the phylogenetic tree was generated using IQTREE (v.1.6.12) with 100 bootstraps and a GTR + I + G model. Established paramyxovirus genera are annotated.

Phylogenetic analysis of the CoV and PMV viral sequences identified was conducted, generating three phylogenetic trees. Within bats positive for a CoV, positive sequences fell into three groups (Figs. 4 and 5). Three different species of insectivorous bats were positive for Eidolon bat coronavirus, a betacoronavirus within the subgenus *Nobecovirus*. The remaining CoV sequences fell into two distinct groups within the genus *Alphacoronavirus* that do not clearly fall into any established subgenus. The PREDICT CoV-114 virus appears phylogenetically closely related to Chaerephon bat coronavirus Kenya/KY22/2006 (GenBank accession number HQ728486); further analysis would be necessary to confirm if they could be considered the same viral species. Within the bats positive for a PMV, positive viral sequences clustered within two genera: *Respirovirus and* the proposed genus *Shaanvirus* (Fig. 6) (Noh et al. 2018; Jang et al. 2020).

## Discussion

This study presents new information on the presence and diversity of coronaviruses and paramyxoviruses in four species of insectivorous bats in Ethiopia. These risk interface sites were selected due to recognized potential for people to come into contact with bats as well as with their excrement, including in buildings and dwellings in urban and peri-urban locations and in a blister cave adjacent to a major transit corridor. All *Rhinopoma hardwickii* bats were from a cave easily accessed by humans that is used for shelter as well as a source for water from the floor of the cave. The cave water is used for activities such as washing container trucks that travel long distances across the Horn of Africa, and thus there is the potential for human exposure to coronaviruses during activities, such as through spraying contaminated water. The remainder of bat sampling sites included roofs of buildings (including residences, primary schools, health clinics, and businesses) in urban city centers, which are also high-risk interfaces for people to contact bats and bat excrement.

There is currently no evidence to suggest that the particular viruses identified in this study pose a significant threat to human health. In this study, bat species were sampled that are known to carry viruses of public health interest, such as Bombali ebolavirus in *Chaerephon pumilus. Chaerephon pumilus* bats have also been previously reported to test positive for SARSr-CoVs in neighboring Kenya (Tong et al. 2009; Waruhiu et al. 2017), which is interesting given the association of these viruses with rhinolophid bats (Anthony et al. 2017b; Wells et al. 2021). While *Rhinopoma harwickii*, or any species from the Rhinopomatidae family, are not known to be hosts of zoonotic coronaviruses, they are in the broader Rhinolophoidea superfamily, which contains a number of species known to be associated with SARSr-CoVs (Lau et al. 2005; Li et al. 2005; Anthony et al. 2017b; Fan et al. 2019; Latinne et al. 2020; Crook et al. 2021; Wacharapluesadee et al. 2021; Wells et al. 2021).

This study identified two paramyxoviruses in nine bats (PREDICT PMV-175, *Mops midas, n* = 7; PREDICT PMV-24, *Chaerephon pumilus, n* = 2) sampled from buildings in Awash and Bati. PREDICT PMV-175 falls within the proposed genus *Shaanvirus*, which expands the known geographical distribution of this genus; to date there are few reports of *Shaanviruses* identified in African bats (Noh et al. 2018; Jang et al. 2020). PREDICT PMV-24 falls within *Respirovirus*, a genus which includes established human and animal pathogens including human and livestock parainfluenza viruses. Both PREDICT PMV-24 positive *Chaerephon pumilus* bats in Awash exhibited co-infections with Chaerephon bat coronavirus/Kenya/KY22/2006. While whole genome sequencing of both paramyxoviruses is necessary for full viral characterization and comparison to other known paramyxoviruses, these findings offer new information on the diversity of paramyxoviruses in insectivorous bat species in Ethiopia.

We found a previously undescribed alphacoronavirus, PREDICT_CoV-114 in nearly half (41.4%) of all *Rhinopoma hardwickii* bats sampled in our study, and one adult female sampled in August 2018 demonstrated co-infection with the betacoronavirus Eidolon bat coronavirus, a phenomenon considered a risk factor for the recombination and natural emergence of novel viral strains (Lau et al. 2010; Ge et al. 2016; Kumar et al. 2018). Researchers in Saudi Arabia previously identified several CoVs in noninvasively collected roost fecal samples from *Rhinopoma hardwickii,* but not from individually sampled animals (Memish et al. 2013). While little data are available on the spatial distribution and life cycle history of *Rhinopoma hardwickii* in Ethiopia, other published reports of the reproductive patterns of the species in the northern hemisphere indicate these bats exhibit a single estrus period per year that results in a birth pulse between June and July (Qumsiyeh and Jones 1986; Karim et al. 1989). Paired with a 20-day lactation cycle, this timing could be consistent with an August peak of increased shedding of PREDICT_CoV-114 by *Rhinopoma hardwickii* (adults and subadults) sampled in the blister cave colony at a time of potentially increased reproductive stress. Recent evidence suggests some CoV shedding may be more likely at the time of weaning from the dam (Montecino-Latorre et al. 2020). The five *Mops midas* bats that also tested positive for PREDICT_CoV-114 were all sampled in August 2018. This host species represents a different family group, sampled from a geographically distinct site and with a different roosting preference. Sparse scientific literature suggests multiple breeding seasons for *Mops midas* bats in other regions of Africa (January–March, Uganda; December–February, Botswana) with a second breeding season may be possible as the same resource reports a lactating female found in October (Kingdon 1974; Smithers 1983). It is challenging to extrapolate breeding seasons across different hemispheres and geographies; the timing of shedding of this virus in relation to the life history and reproductive cycle of *Mops midas* bats in Ethiopia needs further study.

The research team identified PREDICT_CoV-114 virus in *Rhinopoma hardwickii* bats sampled from the roadside blister cave in both 2016 and 2018; in 2016, 27.3% (*n* = 9/33) of bats sampled were positive, and in 2018, 52.5% (*n* = 63/120) of bats sampled were positive for this virus. In 2017, no PREDICT_CoV-114-positive samples were detected in the 21 *Rhinopoma hardwickii* bats sampled in early September. This lack of detection could be due to small sample size (*n* = 21), lack of virus presence, missing a very narrow “seasonal” window of viral shedding for this particular virus/species combination, or other yet to be determined factors. These findings highlight what others hve also recognized: that there may be significant variability in viral shedding differences across bat species, season, roosting location, life history, reproductive cycles, environment, climate, external stressors, virus characteristics, and other factors (Letko et al. 2020; Montecino-Latorre et al. 2020; Kumakamba et al. 2021; Joffrin et al. 2022).

The detection of the PREDICT_CoV-114 virus in samples collected almost exclusively during the primary wet season follows the trend reported by some papers (Lau et al. 2010; Maganga et al. 2020), but diverges from other published results (Anthony et al. 2017b; Valitutto et al. 2020; Kumakamba et al 2021.) This study used the TerraClimate dataset to assign “wet” or “dry” season to sampling events. When triangulated with historical knowledge and on the ground reports of “seasons” by local residents, this method may be stronger for classifying season than relying exclusively on local reporting of the months typically associated with a “wet” or “dry” season, simple geographic location/distance from the equator, or other subjective methods of reporting season. As the disease ecology field continues to document and understand viral shedding in different species in environments experiencing climatic shifts and stressors, more rigorous documentation and determination of season are important for improved understanding of the roles that climate, season, food availability, and other environmental factors may have on viral shedding, as well as how these factors are associated with animal lifecycles related to parturition, lactation, and weaning.

This study found the highest proportion of CoV positives in rectal swabs compared to oral swabs from the same animals, a finding consistent with previous studies (Anthony et al. 2017b; Valitutto et al. 2020). This fecal shedding has implications for how to design and perform viral surveillance and interventions to reduce risk for exposure to CoVs in people. Noninvasive surveillance, such as testing guano may be a cost-effective, less invasive way to implement longitudinal screening to better understand the timing of viral shedding and thus better inform individual level sampling efforts as well as the timing of potential risk, as multiple variables likely affect viral shedding. Based on other studies, viruses can be shed during times of stress, such as during pregnancy and weaning (Amman et al. 2012; Montecino-Latorre et al. 2020). Noninvasive disease surveillance efforts in wildlife could be paired with increased use of effective biodiversity monitoring tools, such as sound recording of bat echolocation signals which has the potential to differentiate between species, a method effectively used by a bat census team in the Kafa Biosphere Reserve in Ethiopia, and laser technology (Light Detection and Ranging; LIDAR) to estimate and identify roosting bats (Azmy et al. 2012; NABU 2017). Paired and interpreted together, noninvasive viral surveillance with biodiversity characterization and more accurate species counts could inform more targeted individual surveillance efforts (e.g., by species, by season, by site, etc.).

Our study relied on partial viral fragments for comparison and for identification of clusters of sequences in ‘operational taxonomic units,’ a cost-effective approach previously described and used by other researchers (Anthony et al. 2017a, b; Valitutto et al. 2020). As the next step to begin to characterize these viruses, whole genome sequencing of the positive specimens could be pursued in the future to better classify these virus sequences, which could yield more biological information on the viruses detected in this study. Future surveillance efforts may also consider deep sequencing in combination with the aforementioned noninvasive population ecology methodologies, providing more detailed insights into the diversity of the bat virome in concert with more nuanced species ecology data.

## Conclusion

When considering similar cave and building risk interface settings around the world, improved awareness of viral diversity in bats at the community level is recommended to inform on when, where, and how to reduce contact with wildlife as part of mitigating risks for viral exposure and spillover across hosts. Improved knowledge of the ecology and distribution of bat species, the types of viruses present, timing of viral shedding, and contact with people in Ethiopia, as well as the larger region, will also help guide future surveillance and viral spillover prevention efforts. Given that bats provide essential ecosystem services for pollination and insect control, we must find ways to manage public health risks for community members while protecting bats that fill key ecologic niches.

## Supplementary Information

Below is the link to the electronic supplementary material.

Supplementary file1 (XLSX 19 KB)

## Data Availability

The datasets generated and analyzed during the current study are available on the USAID Development Data Library at https://data.usaid.gov/Global-Health-Security-in-Development-GHSD-/PREDICT-Emerging-Pandemic-Threats-Project/tqea-hwmr. GenBank sequence data are available in the supplemental table.

## Code Availability

Code used for analysis and figure development is available at https://doi.org/10.5281/zenodo.6032454.

## References

[CR1] Abatzoglou JT, Dobrowski SZ, Parks SA, Hegewisch KC (2018). TerraClimate, a high-resolution global dataset of monthly climate and climatic water balance from 1958–2015. Scientific Data.

[CR2] Allen T, Murray KA, Zambrana-Torrelio C, Morse SS, Rondinini C, Di Marco M, Breit N, Olival KJ, Daszak P (2017). Global hotspots and correlates of emerging zoonotic diseases. Nature Communications.

[CR3] Amman BR, Carroll SA, Reed ZD, Sealy TK, Balinandi S, Swanepoel R, Kemp A, Erickson BR, Comer JA, Campbell S, Cannon DL, Khristova ML, Atimnedi P, Paddock CD, Kent Crockett RJ, Flietstra TD, Warfield KL, Unfer R, Katongole-Mbidde E, Downing R, Tappero JW, Zaki SR, Rollin PE, Ksiazek TG, Nichol ST, Towner JS (2012). Seasonal pulses of Marburg virus circulation in juvenile Rousettus aegyptiacus bats coincide with periods of increased risk of human infection. PLoS Pathology.

[CR4] Amman BR, Nyakarahuka L, McElroy AK, Dodd KA, Sealy TK, Schuh AJ, Shoemaker TR, Balinandi S, Atimnedi P, Kaboyo W, Nichol ST (2014). Marburgvirus resurgence in Kitaka Mine bat population after extermination attempts Uganda. Emerging Infectious Diseases.

[CR5] Amman BR, Bird BH, Bakarr IA, Bangura J, Schuh AJ, Johnny J, Sealy TK, Conteh I, Koroma AH, Foday I, Amara E (2020). Isolation of Angola-like Marburg virus from Egyptian rousette bats from West Africa. Nature Communications.

[CR6] Anthony SJ, Ojeda-Flores R, Rico-Chavez O, Navarrete-Macias I, Zambrana-Torrelio CM, Rostal MK, Epstein JH, Tipps T, Liang E, Sanchez-Leon M, Sotomayor-Bonilla J (2012). Coronaviruses in bats from Mexico. Journal of General Virology.

[CR7] Anthony SJ, Leger JS, Pugliares K, Ip HS, Chan JM, Carpenter ZW, Navarrete-Macias I, Sanchez-Leon M, Saliki JT, Pedersen J, Karesh W, Daszak P, Rabadan R, Rowles T, Lipkin WI (2012). Emergence of fatal avian influenza in New England harbor seals. mBio.

[CR8] Anthony SJ, Islam A, Johnson CK, Navarrete-Macias I, Liang E, Jain K, Hitchens PL, Che X, Soloyvov A, Hicks AL, Ojeda-Flores R, Zambrana-Torrelio C, Ulrich W, Rostal MK, Petrosov A, Garcia J, Haider N, Wolfe N, Goldstein T, Morse SS, Rahman M, Epstein JH, Mazet JAK, Daszak P, Lipkin I (2015). Non-random patterns in viral diversity. Nature Communications.

[CR9] Anthony SJ, Gilardi K, Menachery VD, Goldstein T, Ssebide B, Mbabazi R, Navarrete-Macias I, Liang E, Wells H, Hicks A, Petrosov A, Byarugaba DK, Debbink K, Dinnon KH, Scobey T, Randell SH, Yount BL, Cranfield M, Johnson CK, Baric RS, Lipkin WI, Mazet JA (2017). Further Evidence for bats as the evolutionary source of middle east respiratory syndrome coronavirus. mBio.

[CR10] Anthony SJ, Johnson CK, Greig DJ, Kramer S, Che X, Wells H, Hicks AL, Joly DO, Wolfe ND, Daszak P, Karesh W (2017). Global patterns in coronavirus diversity. Virus Evolution.

[CR11] Azmy SN, Sah SAM, Shafie NJ, Ariffin A, Majid Z, Ismail MNA, Shamsir MS (2012). Counting in the dark: Non-intrusive laser scanning for population counting and identifying roosting bats. Scientific Reports.

[CR12] Calisher CH, Childs JE, Field HE, Holmes KV, Schountz T (2006). Bats: important reservoir hosts of emerging viruses. Clinical Microbiology Reviews.

[CR13] Chang, W. (2014). Extrafont: Tools for using fonts. R package version 0.17. Available at: https://CRAN.R-project.org/package=extrafont

[CR14] Changula K, Kajihara M, Mori-Kajihara A, Eto Y, Miyamoto H, Yoshida R, Shigeno A, Hang’ombe B, Qiu Y, Mwizabi D, Squarre D (2018). Seroprevalence of filovirus infection of Rousettus aegyptiacus bats in Zambia. The Journal of Infectious Diseases.

[CR15] Cheung WH, Senay GB, Singh A (2008). Trends and spatial distribution of annual and seasonal rainfall in Ethiopia. International Journal of Climatology: A Journal of the Royal Meteorological Society.

[CR16] Corman VM, Ithete NL, Richards LR, Schoeman MC, Preiser W, Drosten C, Drexler JF (2014). Rooting the phylogenetic tree of middle east respiratory syndrome coronavirus by characterization of a conspecific virus from an African bat. Journal of Virology.

[CR17] Corman VM, Kallies R, Philipps H, Göpner G, Müller MA, Eckerle I, Brünink S, Drosten C, Drexler JF (2014). Characterization of a novel betacoronavirus related to middle East respiratory syndrome coronavirus in European hedgehogs. Journal of Virology.

[CR18] Corman VM, Landt O, Kaiser M, Molenkamp R, Meijer A, Chu DK, Bleicker T, Brünink S, Schneider J, Schmidt ML, Mulders DG, Haagmans BL, van der Veer B, van den Brink S, Wijsman L, Goderski G, Romette JL, Ellis J, Zambon M, Peiris M, Goossens H, Reusken C, Koopmans MP, Drosten C (2020). Detection of 2019 novel coronavirus (2019-nCoV) by real-time RT-PCR. Eurosurveillance.

[CR19] Crook JM, Murphy I, Carter DP, Pullan ST, Carroll M, Vipond R, Cunningham AA, Bell D (2021). Metagenomic identification of a new sarbecovirus from horseshoe bats in Europe. Scientific Reports.

[CR20] Fan Y, Zhao K, Shi ZL, Zhou P (2019). Bat coronaviruses in China. Viruses.

[CR21] Firke, S. (2020). Janitor: Simple tools for examining and cleaning dirty data. R package version 2.0.1. Available at: https://CRAN.R-project.org/package=janitor

[CR22] Food and Agriculture Organization of the United Nations. (2011). Investigating the role of bats in emerging zoonoses: Balancing ecology, conservation and public health interests. Edited by S.H. Newman, H.E. Field, C.E. de Jong and J.H. Epstein. *FAO Animal Production and Health Manual No. 12.* Rome. Available at: http://www.fao.org/3/a-i2407e.pdf

[CR23] Forbes KM, Webala PW, Jääskeläinen AJ, Abdurahman S, Ogola J, Masika MM, Kivistö I, Alburkat H, Plyusnin I, Levanov L, Korhonen EM, Huhtamo E, Mwaengo D, Smura T, Mirazimi A, Anzala O, Vapalathi O, Sironen T (2019). Bombali virus in Mops condylurus bat Kenya. Emerging Infectious Diseases.

[CR24] Goldstein T, Anthony SJ, Gbakima A, Bird BH, Bangura J, Tremeau-Bravard A, Belaganahalli MN, Wells HL, Dhanota JK, Liang E, Grodus M (2018). The discovery of Bombali virus adds further support for bats as hosts of ebolaviruses. Nature Microbiology.

[CR25] Han BA, Schmidt JP, Alexander LW, Bowden SE, Hayman DT, Drake JM (2016). Undiscovered bat hosts of filoviruses. PLoS Neglected Tropical Diseases.

[CR26] Harrison, E., Drake, T., Ots, R. (2020). finalfit: Quickly Create Elegant Regression Results Tables and Plots when Modelling. R package version 1.0.0. Available at: https://CRAN.R-project.org/package=finalfit

[CR27] Jang, S. S., Noh, J. Y., Lo, V. T., Choi, Y. G., Yoon, S.-W., Jeong, D. G., Kim, H. K. (2020). The Epidemiological Characteristics of the Korean Bat Paramyxovirus between 2016 and 2019. *Microorganisms*, *8*(6), 844. MDPI AG. DOI: 10.3390/microorganisms8060844 10.3390/microorganisms8060844 PMC7356101 32512880

[CR28] Joffrin L, Hoarau AO, Lagadec E, Torrontegi O, Köster M, Le Minter G, Dietrich M, Mavingui P, Lebarbenchon C (2022). Seasonality of coronavirus shedding in tropical bats. Royal Society Open Science.

[CR29] Johnson CK, Hitchens PL, Pandit PS, Rushmore J, Evans TS, Young CC, Doyle MM (2020). Global shifts in mammalian population trends reveal key predictors of virus spillover risk. Proceedings of the Royal Society.

[CR30] Kajihara M, Hang’ombe BM, Changula K, Harima H, Isono M, Okuya K, Yoshida R, Mori-Kajihara A, Eto Y, Orba Y, Ogawa H (2019). Marburg virus in Egyptian fruit bats, Zambia. Emerging Infectious Diseases.

[CR31] Karim KB, Banerjee S (1989). Reproduction in the Indian mouse-tailed bat, Rhinopoma hardwickei hardwickei (Chiroptera, Rhinopomatidae). Reproduction, Fertility and Development.

[CR32] Kearse M, Moir R, Wilson A, Stones-Havas S, Cheung M, Sturrock S, Buxton S, Cooper A, Markowitz S, Duran C, Thierer T, Ashton B, Meintjes P, Drummond A (2012). Geneious basic: an integrated and extendable desktop software platform for the organization and analysis of sequence data. Bioinformatics.

[CR33] Kingdon, J. (1974). East African Mammals: an atlas of evolution in Africa. Academic Press, London, 2A: I-341.

[CR34] Kreuder Johnson C, Hitchens PL, Smiley Evans T, Goldstein T, Thomas K, Clements A, Joly DO, Wolfe ND, Dasak P, Karesh WB, Mazet JK (2015). Spillover and pandemic properties of zoonotic viruses with high host plasticity. Scientific Reports.

[CR35] Kumakamba C, Niama FR, Muyembe F, Mombouli JV, Kingebeni PM, Nina RA, Lukusa IN, Bounga G, N’Kawa F, Nkoua CG, Atibu Losoma J, Mulembakani P, Makuwa M, Tamufe U, Gillis A, LeBreton M, Olson SH, Cameron K, Reed P, Ondzie A, Tremeau-Bravard A, Smith BR, Pante J, Schneider BS, McIver DJ, Ayukekbong JA, Hoff NA, Rimoin AW, Laudisoit A, Monagin C, Goldstein T, Joly DO, Saylors K, Wolfe ND, Rubin EM, MPassi, R.B., Muyembe Tamfum, J.J., Lange, C.E. (2021). Coronavirus surveillance in wildlife from two Congo basin countries detects RNA of multiple species circulating in bats and rodents. PloS One.

[CR36] Kumar N, Sharma S, Barua S, Tripathi BN, Rouse BT (2018). Virological and immunological outcomes of coinfections. Clinical Microbiology Reviews.

[CR37] Kunz TH, Braun de Torrez E, Bauer D, Lobova T, Fleming TH (2011). Ecosystem services provided by bats. Annals of the New York Academy of Sciences.

[CR38] Kuzmin IV, Niezgoda M, Franka R, Agwanda B, Markotter W, Breiman RF, Shieh WJ, Zaki SR, Rupprecht CE (2010). Marburg virus in fruit bat, Kenya. Emerging Infectious Diseases.

[CR39] Latinne A, Hu B, Olival KJ, Zhu G, Zhang L, Li H, Chmura AA, Field HE, Zambrana-Torrelio C, Epstein JH, Li B, Zhang W, Wang LF, Shi ZL, Daszak P (2020). Origin and cross-species transmission of bat coronaviruses in China. Nature Communications.

[CR40] Lau SK, Woo PC, Li KS, Huang Y, Tsoi HW, Wong BH, Wong SS, Leung SY, Chan KH, Yuen KY (2005). Severe acute respiratory syndrome coronavirus-like virus in Chinese horseshoe bats. Proceedings of the National Academy of Sciences of the United States of America.

[CR41] Lau SK, Li KS, Huang Y, Shek CT, Tse H, Wang M, Choi GK, Xu H, Lam CS, Guo R, Chan KH, Zheng BJ, Woo PC, Yuen KY (2010). Ecoepidemiology and complete genome comparison of different strains of severe acute respiratory syndrome-related Rhinolophus bat coronavirus in China reveal bats as a reservoir for acute, self-limiting infection that allows recombination events. Journal of Virology.

[CR42] Letko M, Seifert SN, Olival KJ, Plowright RK, Munster VJ (2020). Bat-borne virus diversity, spillover and emergence. Nature Reviews Microbiology.

[CR43] Li W, Shi Z, Yu M, Ren W, Smith C, Epstein JH, Wang H, Crameri G, Hu Z, Zhang H, Zhang J (2005). Bats are natural reservoirs of SARS-like coronaviruses. Science.

[CR44] Lüdecke D, Ben-Shachar M, Patil I, Makowski D (2020). Extracting, computing and exploring the parameters of statistical models using R. Journal of Open Source Software.

[CR45] Maganga GD, Pinto A, Mombo IM, Madjitobaye M, Beyeme AMM, Boundenga L, Gouilh MA, N’Dilimabaka N, Drexler JF, Drosten C, Leroy EM (2020). Genetic diversity and ecology of coronaviruses hosted by cave-dwelling bats in Gabon. Scientific Reports.

[CR46] Markotter W, Geldenhuys M, Jansen van Vuren P, Kemp A, Mortlock M, Mudakikwa A, Nel L, Nziza J, Paweska J, Weyer J (2019). Paramyxo-and coronaviruses in Rwandan bats. Tropical Medicine and Infectious Disease.

[CR47] Memish ZA, Mishra N, Olival KJ, Fagbo SF, Kapoor V, Epstein JH, AlHakeem R, Durosinloun A, Al Asmari M, Islam A, Kapoor A, Briese T, Dasak P, Al Rabeeah AA, Lipkin W (2013). Middle east respiratory syndrome coronavirus in bats Saudi Arabia. Emerging Infectious Diseases.

[CR48] Menzel M.A., Menzel, J.M., Castleberry, S. B., Ozier, J., Ford, W.M., Edwards, J.W. (2002). *Illustrated Key to Skins and Skulls of Bats in the Southeastern and Mid-Atlantic States.* USDA Forest Service, PA. Available at: https://www.fs.fed.us/ne/newtown_square/publications/research_notes/pdfs/2002/rnne376.pdf

[CR49] Minh BQ, Schmidt HA, Chernomor O, Schrempf D, Woodhams MD, von Haeseler A, Lanfear R (2020). IQ-TREE 2: New models and efficient methods for phylogenetic inference in the genomic era. Molecular Biology and Evolution.

[CR50] Montecino-Latorre D, Goldstein T, Gilardi K, Wolking D, Van Wormer E, Kazwala R, Ssebide B, Nziza J, Sijali Z, Cranfield M, Mazet JA (2020). Reproduction of East-African bats may guide risk mitigation for coronavirus spillover. One Health Outlook.

[CR51] Moureau G, Temmam S, Gonzalez JP, Charrel RN, Grard G, De Lamballerie X (2007). A real-time RT-PCR method for the universal detection and identification of flaviviruses. Vector-Borne and Zoonotic Diseases.

[CR53] Noh JY, Jeong DG, Yoon SW, Kim JH, Choi YG, Kang SY, Kim HK (2018). Isolation and characterization of novel bat paramyxovirus B16–40 potentially belonging to the proposed genus Shaanvirus. Scientific Reports.

[CR54] Nziza J, Goldstein T, Cranfield M, Webala P, Nsengimana O, Nyatanyi T, Mudakikwa A, Tremeau-Bravard A, Byarugaba D, Tumushime JC, Mwikarago IE, Mazet J, Gilardi K (2020). Coronaviruses detected in bats in close contact with humans in Rwanda. Ecohealth.

[CR56] Plowright RK, Parrish CR, McCallum H, Hudson PJ, Ko AI, Graham AL, Lloyd-Smith JO (2017). Pathways to zoonotic spillover. Nature Reviews Microbiology.

[CR57] PREDICT Consortium. (2016). PREDICT Operating Procedures 5.2.7: Bat Sampling Methods. Available at: https://ohi.sf.ucdavis.edu/sites/g/files/dgvnsk5251/files/files/page/predict-sop-bat-sampling-2017.pdf

[CR58] Pourrut X, Souris M, Towner JS, Rollin PE, Nichol ST, Gonzalez JP, Leroy E (2009). Large serological survey showing cocirculation of Ebola and Marburg viruses in Gabonese bat populations, and a high seroprevalence of both viruses in Rousettus aegyptiacus. BMC Infectious Diseases.

[CR59] Quan PL, Firth C, Street C, Henriquez JA, Petrosov A, Tashmukhamedova A, Hutchison SK, Egholm M, Osinubi MO, Niezgoda M, Ogunkoya AB, Briese T, Rupprecht CE, Lipkin WI (2010). Identification of a severe acute respiratory syndrome coronavirus-like virus in a leaf-nosed bat in Nigeria. Mbio.

[CR62] Qumsiyeh MB, Jones JK (1986). Rhinopoma hardwickii and Rhinopoma muscatellum. Mammalian Species.

[CR60] R Core Team (2019). R: A language and environment for statistical computing. R Foundation for Statistical Computing, Vienna, Austria. Available at: https://www.R-project.org/

[CR61] Rudis, B. (2019). hrbrthemes: Additional Themes, Theme Components and Utilities for 'ggplot2'. R package version 0.6.0. Available at https://CRAN.R-project.org/package=hrbrthemes

[CR63] Smithers, R. H. N. (1983). The mammals of the southern Africa subregion. University of Pretoria, Pretoria, Republic of South Africa, 736 pp.

[CR52] The Nature and Biodiversity Conservation Union (NABU) (eds.), 2017: NABU’s Biodiversity Assessment at the Kafa Biosphere Reserve. Berlin, Addis Ababa. Available at: http://imperia.verbandsnetz.nabu.de/imperia/md/content/nabude/international/nabu_biodiversity_assessment_15.pdf

[CR64] Tong S, Chern SWW, Li Y, Pallansch MA, Anderson LJ (2008). Sensitive and broadly reactive reverse transcription-PCR assays to detect novel paramyxoviruses. Journal of Clinical Microbiology.

[CR65] Tong S, Conrardy C, Ruone S, Kuzmin IV, Guo X, Tao Y, Niezgoda M, Haynes L, Agwanda B, Breiman RF, Anderson LJ, Rupprecht CE (2009). Detection of novel SARS-like and other coronaviruses in bats from Kenya. Emerging Infectious Diseases.

[CR66] Towner JS, Pourrut X, Albariño CG, Nkogue CN, Bird BH, Grard G, Ksiazek TG, Gonzalez JP, Nichol ST, Leroy EM (2007). Marburg virus infection detected in a common African bat. PLoS One.

[CR67] Towner JS, Amman BR, Sealy TK, Carroll SAR, Comer JA, Kemp A, Swanepoel R, Paddock CD, Balinandi S, Khristova ML, Formenty PB (2009). Isolation of genetically diverse Marburg viruses from Egyptian fruit bats. PLoS Pathology.

[CR68] Townzen JS, Brower AVZ, Judd DD (2008). Identification of mosquito bloodmeals using mitochondrial cytochrome oxidase subunit I and cytochrome b gene sequences. Medical and Veterinary Entomology.

[CR55] van Paweska JT, Vuren PJ, Kemp A, Storm N, Grobbelaar AA, Wiley MR, Palacios G, Markotter W (2018). Marburg virus infection in Egyptian rousette bats, South Africa, 2013–2014. Emerging Infectious Diseases.

[CR69] Vijaykrishna D, Smith GJ, Zhang JX, Peiris JSM, Chen H, Guan Y (2007). Evolutionary insights into the ecology of coronaviruses. Journal of Virology.

[CR70] Voigt CC, Kingston T (2016). Bats in the Anthropocene: Conservation of bats in a changing world.

[CR71] Wacharapluesadee S, Duengkae P, Rodpan A, Kaewpom T, Maneeorn P, Kanchanasaka B, Yingsakmongkon S, Sittidetboripat N, Chareesaen C, Khlangsap N, Pidthong A (2015). Diversity of coronavirus in bats from Eastern Thailand. Virology Journal.

[CR72] Wacharapluesadee S, Tan CW, Maneeorn P, Duengkae P, Zhu F, Joyjinda Y, Kaewpom T, Chia WN, Ampoot W, Lim BL, Worachotsueptrakun K, Chen VC, Sirichan N, Ruchisrisarod C, Rodpan A, Noradechanon K, Phaichana T, Jantarat N, Thongnumchaima B, Tu C, Crameri G, Stokes MM, Hemachudha T, Wang LF (2021). Evidence for SARS-CoV-2 related coronaviruses circulating in bats and pangolins in Southeast Asia. Nature Communications.

[CR73] Waruhiu C, Ommeh S, Obanda V, Agwanda B, Gakuya F, Ge XY, Yang XL, Wu LJ, Zohaib A, Hu B, Shi ZL (2017). Molecular detection of viruses in Kenyan bats and discovery of novel astroviruses, caliciviruses and rotaviruses. Virologica Sinica.

[CR74] Watanabe S, Masangkay JS, Nagata N, Morikawa S, Mizutani T, Fukushi S, Alviola P, Omatsu T, Ueda N, Iha K, Taniguchi S, Fujii H, Tsuda S, Endoh M, Kato K, Tohya Y, Kyuwa S, Yoshikawa Y, Akashi H (2010). Bat coronaviruses and experimental infection of bats, the Philippines. Emerging Infectious Diseases.

[CR75] Wells HL, Letko M, Lasso G, Ssebide B, Nziza J, Byarugaba DK, Navarrete-Macias I, Liang E, Cranfield M, Han BA, Tingley MW, Diuk-Wasser M, Goldstein T, Johnson CK, Mazet J, Chandran K, Munster VJ, Gilardi K, Anthony SJ (2021). The evolutionary history of ACE2 usage within the coronavirus subgenus Sarbecovirus. Virus Evolution.

[CR76] Wickham H (2016). ggplot2: Elegant graphics for data analysis.

[CR77] Wickham, H. (2019a). forcats: Tools for Working with Categorical Variables (Factors). R package version 0.4.0. Available at: https://CRAN.R-project.org/package=forcats

[CR78] Wickham, H. (2019b). stringr: Simple, Consistent Wrappers for Common String Operations. R package version 1.4.0. Available at: https://CRAN.R-project.org/package=stringr

[CR79] Wickham, H., François, R., Henry, L., Müller, K. (2020). dplyr: A Grammar of Data Manipulation. R package version 0.8.4. Available at: https://CRAN.R-project.org/package=dplyr

[CR80] Wickham, H., Henry, L. (2020). tidyr: Tidy Messy Data. R package version 1.0.2. Available at: https://CRAN.R-project.org/package=tidyr

[CR81] Wilke, C.O. (2019). cowplot: Streamlined Plot Theme and Plot Annotations for 'ggplot2'. R package version 1.0.0. Available at: https://CRAN.R-project.org/package=cowplot

[CR82] Woo PC, Lau SK, Li KS, Poon RW, Wong BH, Tsoi HW, Yip BC, Huang Y, Chan KH, Yuen KY (2006). Molecular diversity of coronaviruses in bats. Virology.

[CR83] Yob JM, Field H, Rashdi AM, Morrissy C, van der Heide B, Rota P, bin Adzhar A, White J, Daniels P, Jamaluddin A, Ksiazek T (2001). Nipah virus infection in bats (order Chiroptera) in peninsular Malaysia. Emerging Infectious Diseases.

[CR84] Zhai J, Palacios G, Towner JS, Jabado O, Kapoor V, Venter M, Grolla A, Briese T, Paweska J, Swanepoel R, Feldmann H, Nichol ST, Lipkin WI (2007). Rapid molecular strategy for filovirus detection and characterization. Journal of Clinical Microbiology.

[CR85] Zhou P, Yang XL, Wang XG, Hu B, Zhang L, Zhang W, Si HR, Zhu Y, Li B, Huang CL, Chen HD, Chen J, Luo Y, Guo H, Jiang RD, Liu MQ, Chen Y, Shen XR, Wang X, Zheng XS, Zhao K, Chen QJ, Deng F, Liu LL, Yan B, Zhan FX, Wang YY, Xiao GF, Shi ZL (2020). A pneumonia outbreak associated with a new coronavirus of probable bat origin. Nature.

